# Physiological Phenomenology of Neurally-Mediated Syncope with Management Implications

**DOI:** 10.1371/journal.pone.0026489

**Published:** 2011-10-25

**Authors:** Christoph Schroeder, Jens Tank, Karsten Heusser, André Diedrich, Friedrich C. Luft, Jens Jordan

**Affiliations:** 1 Institute for Clinical Pharmacology, Hannover Medical School, Hannover, Germany; 2 Experimental Clinical Research Center, Medical University Charité and Max-Delbrueck Center for Molecular Medicine, Berlin, Germany; 3 Division of Clinical Pharmacology, Department of Medicine, Autonomic Dysfunction Center, Vanderbilt University, Nashville, Tennessee, United States of America; Heart Center Munich, Germany

## Abstract

**Background:**

Due to lack of efficacy in recent trials, current guidelines for the treatment of neurally-mediated (vasovagal) syncope do not promote cardiac pacemaker implantation. However, the finding of asystole during head-up tilt –induced (pre)syncope may lead to excessive cardioinhibitory syncope diagnosis and treatment with cardiac pacemakers as blood pressure is often discontinuously measured. Furthermore, physicians may be more inclined to implant cardiac pacemakers in older patients. We hypothesized that true cardioinhibitory syncope in which the decrease in heart rate precedes the fall in blood pressure is a very rare finding which might explain the lack of efficacy of pacemakers in neurally-mediated syncope.

**Methods:**

We studied 173 consecutive patients referred for unexplained syncope (114 women, 59 men, 42±1 years, 17±2 syncopal episodes). All had experienced (pre)syncope during head-up tilt testing followed by additional lower body negative suction. We classified hemodynamic responses according to the modified Vasovagal Syncope International Study (VASIS) classification as mixed response (VASIS I), cardioinhibitory without (VASIS IIa) or with asystole (VASIS IIb), and vasodepressor (VASIS III). Then, we defined the exact temporal relationship between hypotension and bradycardia to identify patients with true cardioinhibitory syncope.

**Results:**

Of the (pre)syncopal events during tilt testing, 63% were classified as VASIS I, 6% as VASIS IIb, 2% as VASIS IIa, and 29% as VASIS III. Cardioinhibitory responses (VASIS class II) progressively decreased from the youngest to the oldest age quartile. With more detailed temporal analysis, blood pressure reduction preceded the heart-rate decrease in all but six individuals (97%) overall and in 10 out of 11 patients with asystole (VASIS IIb).

**Conclusions:**

Hypotension precedes bradycardia onset during head-up tilt-induced (pre)syncope in the vast majority of patients, even in those classified as cardioinhibitory syncope according to the modified VASIS classification. Furthermore, cardioinhibitory syncope becomes less frequent with increasing age.

## Introduction

Neurally-mediated (vasovagal) syncope is common in all age groups [1]–[3]. Patients with frequent syncope have a markedly reduced quality of life, similar to that of patients with severe rheumatoid arthritis or chronic low back pain [4]. Neurally-mediated syncope is induced by sudden vasodilation due to sympathetic nervous system withdrawal with or without vagally-mediated bradycardia or frank asystole. The cause is unknown [5]. Treatment options for highly symptomatic patients not responding to volume loading and physical countermeasures [6], [7] are limited [2]. Almost 100 years ago, Sir Thomas Lewis commented: “Undoubtedly the main cause lies in the blood vessels. Atropine, while raising heart rate leaves the blood pressure below normal and the patient still pale and not fully conscious […]” [8]. Indeed, prevention of bradycardia and asystole with cardiac pacemakers was largely ineffective [9], [10] and is only recommended when severe spontaneous bradycardia is detected during prolonged EKG monitoring, but not on tilt test results alone [11]. Nevertheless, pacemaker treatment is still being investigated in patients with high probability of cardioinhibitory syncope [12] and in our experience a significant proportion of syncope patients undergo cardiac pacemaker implantation outside of clinical trials. Furthermore, the increasing use of implantable EKG loop recorders and head-up tilt testing without continuous measurement of blood pressure may lead to excessive cardioinhibitory syncope diagnosis and treatment. We hypothesized that true cardioinhibitory syncope in which the decrease in heart rate coincides or precedes the fall in blood pressure is rare which would in part explain the lack of efficacy of cardiac pacing in neurally-mediated syncope. We therefore analyzed heart rate and blood pressure responses during head-up tilt induced (pre)syncope to define the temporal relationship between hypotension and bradycardia or asystole and to identify patients with true cardioinhibitory syncope. We were particularly interested in influences of age on these responses because physicians may be more inclined to implant cardiac pacemakers in older patients.

## Methods

### Ethics statement

We conducted a retrospective analysis of data acquired during routine diagnostic work-up in patients with unexplained syncope. Thus, patients did not undergo experimental diagnostic tests or treatments, which would require obtaining a separate informed consent. The analysis of data is covered by the general contract governing medical treatment of the Medical University Charité which all patients signed and agreed on. The institutional ethics committee of the Medical University Charité approved the study analysis and publication of anonymous data.

### Patients

We retrospectively analyzed data of 173 consecutive patients (114 women, 59 men) who were referred to the Autonomic Dysfunction Laboratory of the Medical University Charité for head-up tilt testing as part of the routine diagnostic work-up for unexplained syncope. Only patients who experienced neurally-mediated (pre)syncope during head-up tilt were included in the analysis. Patients (n = 132) either received no current medications or their medications were discontinued at least 5 half-lives before testing. In 41 patients, medication had not been fully discontinued.

### Head-up tilt protocol

Head-up tilt testing began in the morning in the majority of patients (median 10:06 a.m., range 08:43 a.m.–15:21 p.m.). For baseline recordings, patients rested supine in a quiet laboratory at an ambient temperature of 22–23°C. After a stable baseline was achieved, patients were passively tilted to 60° head-up tilt. After 20 min and while still at 60° head-up tilt, lower body negative pressure of −20 mmHg was applied for 10 min, followed by another 10 min at −40 mmHg (Leeds protocol [13]). The tilt test was aborted when finger blood pressure and/or heart rate decreased below baseline values and patients reported symptoms of imminent syncope, such as nausea, dizziness, warmth, visual or hearing difficulties. Throughout the test, heart rate was electrocardiographically monitored (Cardioscreen, Medis, Germany). Brachial blood pressure was measured every 2 min with an automated oscillometric device (Dinamap, Critikon, USA). Beat-to-beat blood pressure was continuously monitored by a finger servo-plethysmomanometer (2300 Finapres, Ohmeda, USA) that was kept at heart level throughout the tilt study.

### Data acquisition and analysis

**Figure 1:**
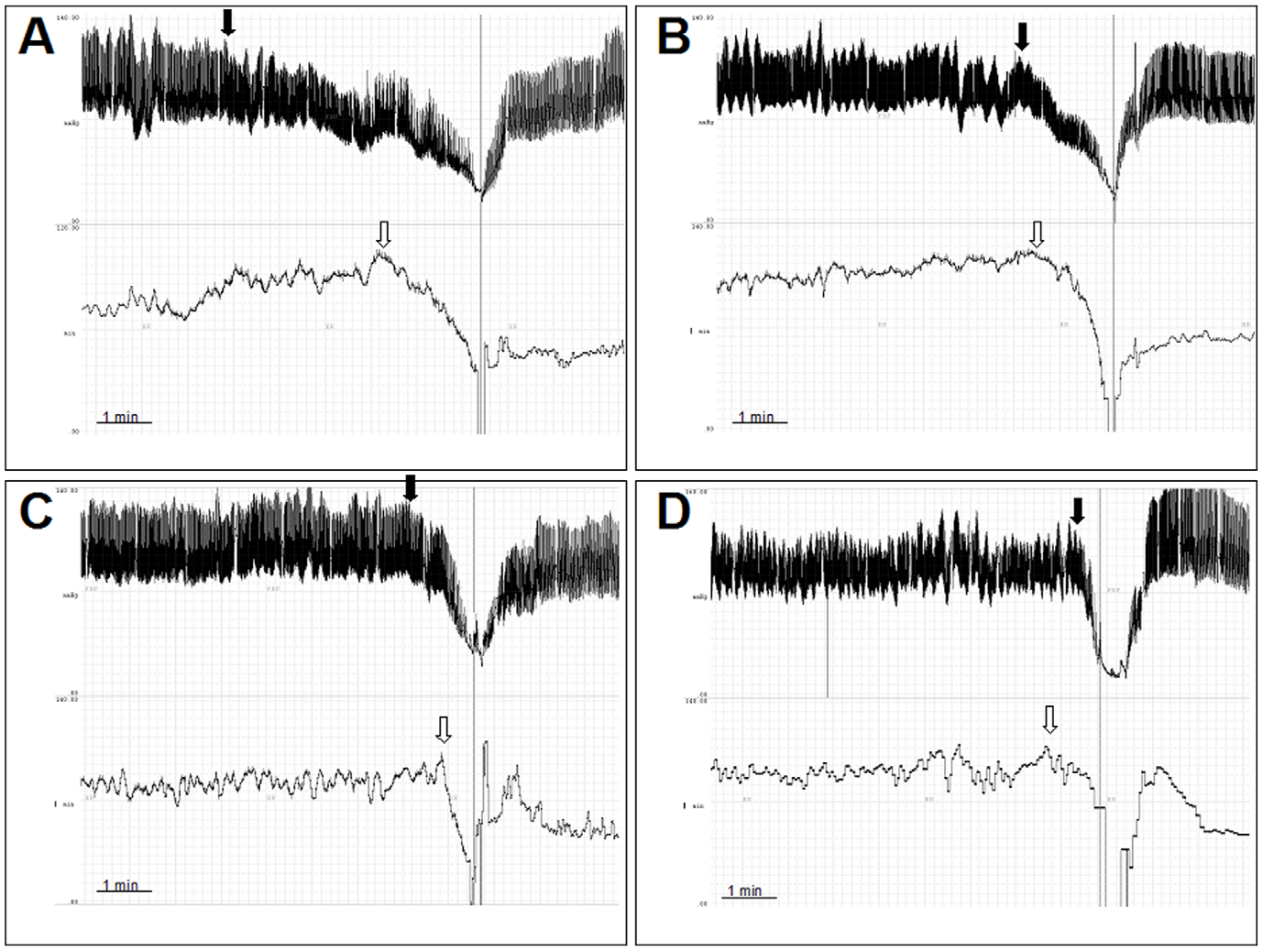
Original tracings of blood pressure (upper tracings) and heart rate (lower tracings) in four representative individuals who experienced a cardioinhibitory response with asystole (VASIS class IIb) during head-up tilt. Black and white arrows indicate the onset of hypotension and bradycardia, respectively. Hypotension preceded the onset of bradycardia in most (Panels A–C) but not all patients (Panel D). Panel A: female, 33 years; panel B: female, 52 years; panel C: female 17 years; panel D: female, 32 years.

Electrocardiogram, finger blood pressure and thoracic impedance signals were analog to digital converted at 500 Hz using the Windaq pro+ software (Dataq Instruments Inc., USA). RR intervals (time between subsequent R waves in the EKG) and blood pressure were defined off-line using a program written by André Diedrich based on PV-wave software (Visual Numerics Inc., USA). The onset of the decrease in blood pressure and – if present – heart rate was identified manually from beat-to-beat recordings (see Figure 1 for examples). Accordingly, blood pressure and heart rate before the onset of (pre)syncope and immediately before termination of the head-up tilt test were determined from beat-to-beat recordings (10 sec mean values). Hemodynamic patterns during (pre)syncope were analyzed according to the modified Vasovagal Syncope International Study (VASIS) classification [14], [15] (Table 1). Furthermore, we calculated the respective slopes of the decrease in blood pressure (and heart rate, if present) by dividing the absolute amount of decrease by the time between the onset of the decrease and the end of head-up tilt. Orthostatic tolerance was assessed as the time from the beginning of head-up tilt to tilt-induced (pre)syncope. The percentage of differences between successive RR intervals greater than 50 ms (pnn50) as a standard index of vagal modulation of heart rate [16] was analyzed from 5 min EKG recordings in the supine position. Patients with atrial fibrillation or multiple atrial or ventricular premature beats were excluded from this analysis.

**Table 1:** Modified VASIS classification [15].

Class	Description	Definition
I	mixed	Decrease in heart rate >10%, minimal heart rate >40 bpm or less than 40 bpm for less than 10 sec with or without asystole of less than 3 sec.Blood pressure falls before heart rate.
IIa	cardioinhibitory without asystole	Minimal heart rate <40 bpm for >10 sec, but asystole of more than 3 sec does not occur.Blood pressure falls before heart rate.
IIb	cardioinhibitory with asystole	Asystole occurs for more than 3 sec.Heart rate coincides with or precedes blood pressure fall.
III	vasodepressor	Decrease in heart rate <10% of maximal heart rate.

### Statistics

All data is expressed as mean±SEM. We compared variances between the groups by using the F test. Differences in parametric data were compared by unpaired t test. Nonparametric data was analyzed by Mann-Whitney U test. For multiple comparisons of continuous data between age quartiles, one-way ANOVA and Bonferroni post-test were applied. Distribution of VASIS classes between groups was analyzed by chi square test. A value for p<.05 was considered significant. All calculations were done with Prism 5.01 (GraphPad Software Inc., USA).

## Results

Mean age was similar in male and female syncope patients (45±2 vs. 41±2 years, ns). Women reported more syncopal episodes (20±3 vs. 11±3, p<.05 over a longer duration than men (11±1 vs. 6±1 years, p<.05), indicating an earlier onset of syncope in women. However, the frequency of syncopal spells was similar in women and in men. Cardiovagal tone assessed by heart rate variability was not different between sexes, but women had lower blood pressure and higher heart rates than men, both in the supine position and throughout head-up tilt. However, orthostatic tolerance did not differ between sexes (women: 1670±39 sec, men: 1711±70 sec, ns) nor between age quartiles (Table 2).

**Table 2:** Clinical data of patients - age quartiles.

		1. Quartile	2. Quartile	3.Quartile	4. Quartile	
		n = 43	n = 44	n = 44	n = 42	
	Unit	Mean±SEM	Mean±SEM	Mean±SEM	Mean±SEM	p
Female gender	[n (%)]	32 (74%)	27 (61%)	29 (66%)	26 (62%)	ns
Age	[years]	19±1	35±1	51±1	66±1	<.001
Age range	[years]	12–24	25–42	43–57	58–79	n/a
Height	[cm]	170±1	174±1	169±1	167±1	<.01
Weight	[kg]	65±2	76±2	77±2	73±2	<.001
Body mass index	[kg/m2]	22.2±0.5	25.2±0.5	26.6±0.6	26.0±0.5	<.001
Number of syncopal episodes	18±5	12±2	27±6	12±3	<.05	
Duration of syncope history	[years]	4±1	10±2	12±2	9±2	<.05
Time to (pre)syncope during HUT	[sec]	1647±71	1723±63	1706±83	1658±62	ns

Clinical characteristics of patients separated into age quartiles. Statistical significance between groups was tested by chi square test for categorical data (gender) or ANOVA for multiple comparisons with Bonferroni post test (all other parameters). HUT = head-up tilt.

**Figure 2:**
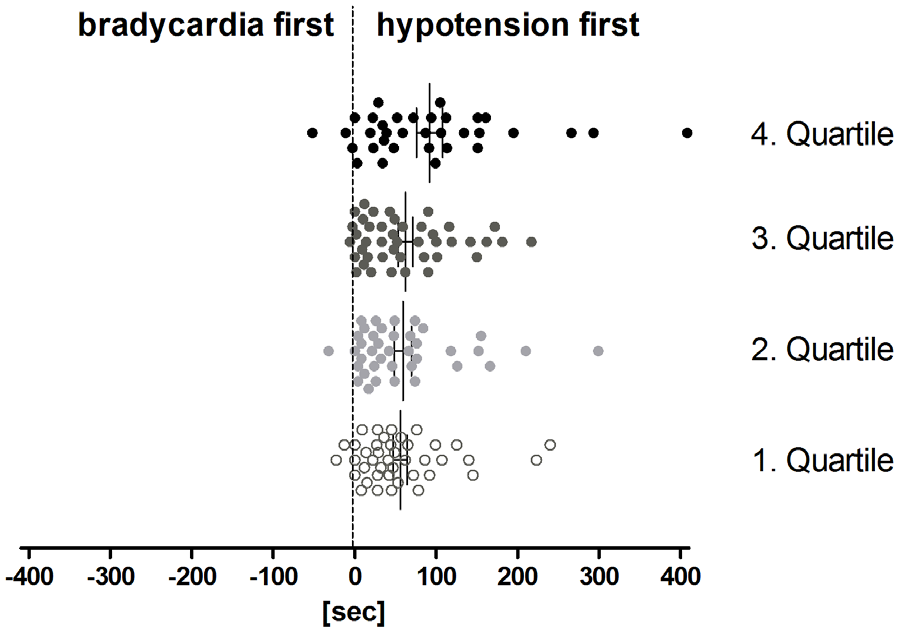
Individual data and mean±SEM in the difference between the onset of hypotension and the onset of bradycardia stratified for age quartiles. Hypotension preceded the decrease in heart rate in all but six individuals (97%) irrespective of age.

**Figure 3:**
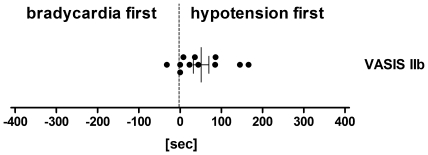
Individual data and mean±SEM in the difference between the onset of hypotension and the onset of bradycardia for those patients who experienced asystole during head-up tilt (VASIS class IIb). Hypotension preceded the decrease in heart rate in all but one individual (91%).

The most common hemodynamic pattern upon tilt-induced (pre)syncope was a mixed response (VASIS class I, 63%), followed by the vasodepressor type (VASIS class III, 29%). Cardioinhibitory with (VASIS IIb, 6%) or without asystole (VASIS IIa, 2%) were much rarer. Interestingly, the onset of hypotension (116±6 sec before (pre)syncope) preceded the onset of bradycardia (47±5 sec) in all but six individuals (97%, Figure 2), irrespective of age. This observation held true for all VASIS groups: even of the eleven patients who presented a cardioinhibitory response with asystole (VASIS IIb) during head-up tilt, the decrease in blood pressure preceded the decrease in heart rate in all but one (91%, Figures 3 and 1, Panel D). Mean duration of asystole was 12.3±2.2 sec (range: 4.4–26.8 sec). Noteworthy, recovery from asystole was spontaneous in all cases, with no other intervention than resuming the supine position.

Clinical data of age quartiles is summarized in Table 3. When stratified for age, the oldest quartile had a lower heart rate in the supine position, but blood pressure did not differ between age quartiles (Table 2). Cardiac vagal tone at rest was successively decreased with increasing age.

**Table 3:** Clinical data of patients.

		All	
		n = 173	
	Unit	Mean±SEM	Range
Female gender	[n (%)]	114 (66%)	n/a
Age	[years]	42±1	12–79
Height	[cm]	170±1	150–194
Weight	[kg]	73±1	46–115
Body mass index	[kg/m2]	25.0±0.3	17.3–35.8
Number of syncopal episodes		17±2	1–120
Duration of syncope history	[years]	9±1	0.1–58
Time to (pre)syncope during HUT	[sec]	1684±35	141–2603

HUT = head-up tilt.

**Figure 4:**
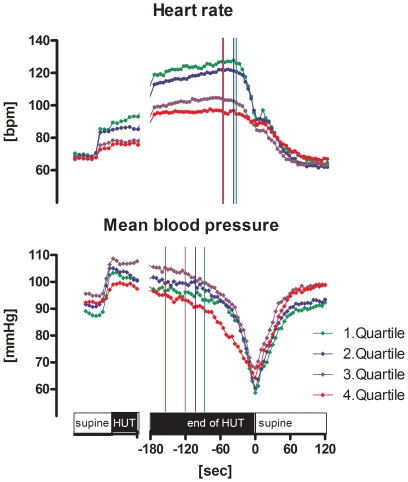
Mean values of heart rate (upper panel) and mean blood pressure (lower panel) at 5 sec intervals in the supine position, during early head-up tilt (HUT), and during tilt-induced (pre)syncope stratified for age (green: 1^st^ quartile, 12–24 years, n = 26; blue: 2^nd^ quartile, 25–42 years, n = 27; violet: 3^rd^ quartile, 43–57 years, n = 36; red: 4^th^ quartile, 58–79 years, n = 20). The vertical lines indicate the onset of decreases in heart rate and blood pressure, respectively. Younger patients had a larger increase in heart rate with head-up tilt. However, the onset of hemodynamic events began earlier with increasing age. Furthermore, the decrease in heart rate upon tilt-induced (pre)syncope was progressively blunted with increasing age.

**Figure 5:**
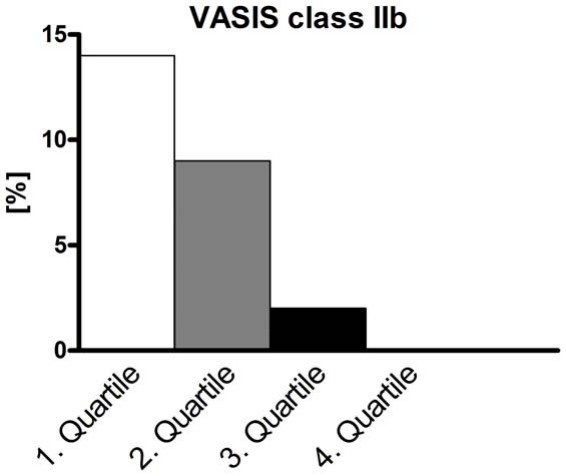
Occurrence of cardioinhibitory responses with asystole (VASIS IIb) for age quartiles. Asystole became progressively rarer with increasing age (p<.05).

With head-up tilt, the increase in heart rate was blunted with age (Figure 4, upper panel). Overall tilt time did not differ between age quartiles. In regard to VASIS classes, cardioinhibitory responses were significantly more frequent with younger age (14% in the youngest quartile compared to 0% in the oldest quartile, p<.05, Table 4 and Figure 5), while vasodepressor responses prevailed with higher age (50% in the oldest quartile compared with 23% in the youngest quartile, p<.01). However, the onset of the decrease in blood pressure and – if present – heart rate upon (pre)syncope came successively earlier with increasing age (Figure 4). Conversely, both, the absolute decreases in blood pressure and heart rate and the slopes of decreases in blood pressure and heart rate were progressively blunted with increasing age. However, absolute values for heart rate and blood pressure immediately at (pre)syncope were not different between age quartiles.

**Table 4:** Hemodynamics at rest and upon (pre)syncope - age quartiles.

		1. Quartile	2. Quartile	3.Quartile	4. Quartile	
		n = 43	n = 44	n = 44	n = 42	
	Unit	Mean±SEM	Mean±SEM	Mean±SEM	Mean±SEM	p
**at supine rest**						
Systolic blood pressure	[mmHg]	121±3	124±3	121±3	130±3	ns
Diastolic blood pressure	[mmHg]	69±2	71±2	70±1	73±2	ns
Heart rate	[bpm]	66±2	70±2	66±1	63±2	<.05
pNN50	[%]	33±3	20±2	11±2	8±2	<.001
**before and at (pre)syncope**						
Onset of BP decrease before (pre)syncope	[sec]	86±9	103±10	120±12	155±15	<.001
MAP before onset of BP decrease	[mmHg]	99±3	101±2	108±3	99±3	ns
HR before onset of BP decrease	[bpm]	124±3	119±4	105±3	96±3	<.001
Onset of HR decrease before (pre)syncope	[sec]	32±3	41±3	59±8	59±10	<.01
MAP before onset of HR decrease	[mmHg]	84±3	90±3	96±3	82±3	<.05
HR before onset of HR decrease	[bpm]	130±3	122±4	107±3	100±4	<.001
MAP at (pre)syncope	[mmHg]	58±3	61±3	67±3	63±3	ns
HR at (pre)syncope	[bpm]	84±7	88±7	81±4	79±5	ns
ΔMAP before (pre)syncope	[mmHg/sec]	−0.7±0.1	−0.6±0.1	−0.5±0.1	−0.3±0.1	<.001
ΔHR before (pre)syncope	[bpm/sec]	−0.5±0.1	−0.4±0.1	−0.2±0.1	−0.1±0.1	<.001
**Modified VASIS class**						p
I	[n (%)]	26 (60%)	27 (61%)	36 (82%)	20 (48%)	<.05
IIa	[n (%)]	1 (2%)	1 (2%)	0 (0%)	1 (2%)	ns#
IIb	[n (%)]	6 (14%)	4 (9%)	1 (2%)	0 (0%)	<.05
III	[n (%)]	10 (23%)	12 (27%)	7 (16%)	21 (50%)	<.01

Statistical significance of differences in continuous and categorical data between age quartiles was tested by ANOVA for multiple comparisons with Bonferroni post test and chi square test, respectively. HUT = head-up tilt.

# = p<.05 for modified VASIS classes IIa and IIb combined.

Modified VASIS class: I = mixed, IIa = cardioinhibitory without asystole, IIb = cardioinhibitory with asystole, III = vasodepressor.

## Discussion

The main finding of our study is that hypotension precedes bradycardia onset during head-up tilt-induced (pre)syncope in the vast majority of patients. The finding is in accordance with earlier studies [17], [18]. Moreover, we observed that blood pressure reduction precedes bradycardia onset even in patients with a cardioinhibitory response according to the modified VASIS classification. Mean blood pressure and heart rate during (pre)syncope were almost identical among age quartiles. Yet, we observed a gradual decline in the proportion of patients with asystole during tilt testing with increasing age.

We applied head-up tilt testing combined with lower body negative pressure to induce neurally-mediated (pre)syncope under laboratory conditions. Compared with standard head-up tilt testing, the methodology may provide a more reproducible measure of orthostatic tolerance [19]. We cannot rule out that the hemodynamic response during head-up tilt testing may differ from the hemodynamic response during spontaneous syncope. A loop electrocardiogram recorder study suggested that the (pre)syncope mechanism during head-up tilt does not correlate well with electrocardiographic findings during an ambulatory episode [20]. In fact, there is conflicting data about the reproducibility of the VASIS type with repeated head-up tilt, which has been reported between 14 and 83% [21]–[24]. Nevertheless, our findings on the physiological phenomenology of neurally-mediated syncope support the notion that the diagnosis of cardioinhibitory type syncope during head-up tilt testing should be scrutinized. In accordance with recent guidelines [11] our findings indicate that head-up tilt test induced cardioinhibition should not be used as indication for cardiac pacemaker implantation.

Occurrence of asystole is disquieting. However, asystole in the setting of neurally-mediated syncope occurs in 1.3–5.1% of adult patients [25]–[28] and up to 10% in children [19]. Yet, mortality of patients with neurally-mediated syncope is not elevated compared to healthy individuals without syncope [29]. Furthermore, asystole during head-up tilt testing does not herald poor outcome in patients with neurally-mediated syncope [27]. Finally, sinus and atrioventricular node function is not impaired in syncope patients who presented with a cardioinhibitory response during head-up tilt compared to those patients of other VASIS classes [30].

Whether or not cardiac pacing may have utility in the treatment of neurally-mediated syncope is still debated. While Sir Thomas Lewis was convinced that vasodilatation and the resulting decrease in peripheral resistance were the prevailing mechanisms [8], recent studies yielded more complex and sometimes conflicting results. Muscle sympathetic activity as a measure of sympathetic vasomotor tone was maintained during tilt-induced (pre)syncope in a patient subset [31]. Furthermore, indirect measurements were consistent with cardiac output reductions during tilt-induced (pre)syncope [32]. Whatever the exact contributions of cardiac output and peripheral resistance to the decrease in blood pressure upon neurally-mediated syncope might be, cardiac pacing counteracts the bradycardia but cannot ameliorate vasodilation. Indeed, cardiac pacing was not effective in neurally-mediated (pre)syncope prevention [18]: while some earlier open-label studies were positive [33]–[35], methodically more sound recent studies were negative [9], [10]. To date, an additional study is still ongoing [12].

In our study, hypotension preceded bradycardia onset in the vast majority of patients including those who presented a cardioinhibitory response with asystole for more than 3 sec. The finding challenges the modified VASIS classification which postulates that in cardioinhibitory syncope with asystole of more than 3 sec the fall in heart rate “coincides with or precedes” the fall in blood pressure [15]. In our study head-up tilt testing was aborted when the decrease in blood pressure became symptomatic. We did not prolong head-up tilt testing until each subject became fully unconscious as it has been proposed by others [15]. Thus, we might have missed some cases of asystole which could have occurred later on. However, we were nevertheless able to observe sequence of the hemodynamic reaction early during (pre)syncope. Furthermore, prolonging head-up tilt could increase the proportion of false-positive cardioinhibitory syncope classifications.

Traditionally, neurally-mediated syncope was considered to primarily affect young people. Recently [3], this notion has been softened possibly due to the increasing use of head-up tilt testing as a diagnostic tool. In 352 patients with unexplained syncope, positive head-up tilt tests occurred in 58% of patients <65 years, but still in 37% and 20% of patients >65 years and >80 years, respectively, with no difference in time to (pre)syncope [36]. In another study in 590 patients with transient loss of consciousness, neurally-mediated syncope was more frequent in younger patients, but still presented the most frequent single cause for syncope even in the oldest group of patients [3]. Other large studies in patients with recurrent syncope found no difference in the rates of positive test results between age groups [25], [37]. In accordance with our findings, the percentage of vasodepressor (VASIS class III) reactions is increasing with age, while cardioinhibitory reactions upon head-up tilt become less frequent with ageing [25], [37], [38]. Thus, older patients with neurally mediated syncope may be even less likely to experience a therapeutic benefit.

The heart rate increase with upright posture decreases with age [39], [40]. In our study, heart rate before the onset of (pre)syncope was also highest in the youngest quartile and decreased with age. Moreover, the rate of heart rate decline during (pre)syncope was blunted with increasing age. These observations suggest cardiac autonomic regulation deteriorates with age. Consistent with earlier studies [41], [42], the percentage of differences between successive RR intervals greater than 50 ms (pNN50) - a measure of cardiac vagal outflow [43] - at rest decreased progressively with age. The decrease in cardiovagal function with ageing could protect from excessive vagal cardioinhibition, thus reducing the proportion of cardioinhibitory responses with or without asystole. Indeed, direct vagal stimulation can provoke asystole in human subjects [44], [45]. Even though cardioinhibitory responses decrease with advancing age, orthostatic tolerance was virtually identical among age groups.

### Clinical implications

We observed that the decrease in blood pressure preceded the decrease in heart rate during (pre)syncope in the vast majority of patients, even in those with cardioinhibitory syncope according to the modified VASIS classification. Furthermore, our findings challenge utility of the modified VASIS classification which is ambivalent in that it requires asystole of more than 3 sec duration and heart rate reduction coinciding or preceding the fall in blood pressure to account for a cardioinhibitory type IIb. As suggested by Sir Thomas Lewis, a decrease in blood pressure rather than a decrease in heart rate initiates the hemodynamic reaction ultimately leading to neurally mediated syncope [8]. One implication of our study is that discontinuous blood pressure monitoring could lead to false diagnostic conclusions. The false diagnosis of cardioinhibitory syncope, which implies that bradycardia precedes hypotension, could then result in unnecessary pacemaker implantations even in the setting of treatment guidelines that state otherwise. Our finding of a clear age-dependency in the occurrence of cardioinhibitory syncope with asystole (VASIS IIb) has possible therapeutic implications for documented episodes of asystole (e.g. during EKG loop recordings): In young individuals asystole frequently occurs during neurally-mediated syncope, representing a transient dysfunction not requiring pacemaker implantation. Conversely, a finding of asystole in patients older than 60 years is unlikely to be explained by neurally-mediated syncope but may rather suggest a structural cardiac disorder.
